# Genetics and Inflammation in Endometriosis: Improving Knowledge for Development of New Pharmacological Strategies

**DOI:** 10.3390/ijms22169033

**Published:** 2021-08-21

**Authors:** Elisa Giacomini, Sabrina Minetto, Letizia Li Piani, Luca Pagliardini, Edgardo Somigliana, Paola Viganò

**Affiliations:** 1 Reproductive Sciences Laboratory, Obstetrics and Gynecology Unit, IRCCS Ospedale San Raffaele, 20132 Milan, Italy; giacomini.elisa@hsr.it (E.G.); pagliardini.luca@hsr.it (L.P.); 2 Obstetrics and Gynecology Unit, IRCCS Ospedale San Raffaele, 20132 Milan, Italy; minetto.sabrina@hsr.it; 3 Department of Clinical Sciences and Community Health, Università degli Studi di Milano, 20122 Milan, Italy; letizia.lipiani@unimi.it (L.L.P.); dadosomigliana@yahoo.it (E.S.); 4 Infertility Unit, Fondazione IRCCS Ca’ Granda, Ospedale Maggiore Policlinico, 20122 Milan, Italy

**Keywords:** endometriosis, inflammation, extracellular vesicles, genetics

## Abstract

According to a rich body of literature, immune cell dysfunctions, both locally and systemically, and an inflammatory environment characterize all forms of endometriosis. Alterations in transcripts and proteins involved in the recruitment of immune cells, in the interaction between cytokines and their receptors, cellular adhesion and apoptosis have been demonstrated in endometriotic lesions. The objective of this narrative review is to provide an overview of the components and mechanisms at the intersection between inflammation and genetics that may constitute vanguard therapeutic approaches in endometriosis. The GWAS technology and pathway-based analysis highlighted the role of the MAPK and the WNT/β-catenin cascades in the pathogenesis of endometriosis. These signaling pathways have been suggested to interfere with the disease establishment via several mechanisms, including apoptosis, migration and angiogenesis. Extracellular vesicle-associated molecules may be not only interesting to explain some aspects of endometriosis progression, but they may also serve as therapeutic regimens per se. Immune/inflammatory dysfunctions have always represented attractive therapeutic targets in endometriosis. These would be even more interesting if genetic evidence supported the involvement of functional pathways at the basis of these alterations. Targeting these dysfunctions through next-generation inhibitors can constitute a therapeutic alternative for endometriosis.

## 1. Endometriosis as a Chronic Inflammatory Disease

The inflammatory nature of endometriosis has been well established over the years [1,2]. A rich body of literature has supported immune cell dysfunctions, both locally and systemically, as being intrinsically linked with the various forms of the disorder, which, in general, consists of endometrial epithelium, stroma, endothelium, immune components and fibrosis in ectopic sites [2,3]. Evidence in this regard has included a paramount role of some immune cells in the lesion establishment and development, an increase of proinflammatory cytokines at peritoneal levels, but also a relevant influence of the disease on systemic immune regulatory molecules [4]. Activated macrophages have received a particular attention, as they have been shown to secrete a panoply of adhesion molecules, growth factors and pro-inflammatory cytokines into the microenvironment of endometriosis lesions and the peritoneal fluid [5,6]. A very recent study on a mouse model of endometriosis has demonstrated multiple origins and different actions for endometriotic lesion-associated macrophages [7]. Lesion-resident macrophages would derive from eutopic endometrium and would promote the growth of the lesions. Conversely, monocyte-derived peritoneal macrophages would protect against the establishment of lesions. Interesting results have also been demonstrated in relation to the recognition of the disease as a local affliction with relevant consequences at the systemic level. Endometriosis is associated with expression of genes in peripheral leukocytes already identified in non-gynaecologic chronic inflammatory disease [8]. Evidence of systemic inflammation in affected women was also supported by the increased proportion of circulating CD141+ myeloid dendritic cells [9].

Causes underlying the development of a chronic inflammation are yet to be completely elucidated, although the retrograde menstrual blood flow as most accepted origin of this disease seems to have a prominent role. As a natural response of innate and adaptive immune system components to try to eliminate menstrual debris, immune cell infiltration and resultant tissue repair are initiated. However, the inability to deal with the persistent presence of menstrual debris over time may lead to an immune system overload and subsequent immune alterations [10,11]. The activational actions of sex steroid hormones that have well-known modulatory effects on the immune responses of young women may predispose to immune-related disorders [12].

Strong support for a causal role of inflammatory pathways in endometriosis establishment derives from the investigation of the genetic factors associated with the disease. Indeed, genome-wide association methodology has allowed the identification of causative variants in/near genes that can be assigned to biological pathways important in chronic inflammation [13]. Immune dysregulation has been reported as a significant contributor to the onset and worsening of the disease, constituting a therapeutic target in endometriosis [14]. Over the years, several studies have been conducted, both in humans and in mice, to evaluate the possible use of immunomodulatory agents in the treatment of endometriosis [15]. Furthermore, genetic evidence supporting the involvement of functional pathways underlying inflammation and immune alterations could be crucial and useful for developing new therapeutic alternatives in endometriosis, where curative treatment is lacking. Indeed, to date, the first-line medical therapy (estrogen/progestin contraceptives or progestin-only contraceptives) is effective in two-thirds of women suffering from endometriosis-related pain [16] and may have limited long-term efficacy. Second-line therapies (injectable depot formulations of gonadotropin-releasing hormone agonists), on the other hand, are associated with menopausal symptoms [17]. Surgery is able to eliminate visible endometriotic lesions but cannot cure the disease and post-operative recurrence is common [18]. Overall, there is a need for effective long-term treatment capable of managing endometriosis symptoms, while alleviating the side effects [19]. This paper focuses on the current knowledge on the research area related to the identification of the immune/inflammatory targets for endometriosis, highlighting novel potential therapeutic approaches.

## 2. From Genetics to Inflammatory Pathways

Studies of the genetic component of endometriosis began as early as 1980 [20], when an increased prevalence of the disease has been observed in first-degree relatives of endometriosis patients compared to controls. A few years later, twin studies demonstrated a sibling genetic relative risk (RR) of 2.34 and a monozygotic/dizygotic (MZ/DZ) ratio of approximately 2 [21], confirming the role of genetics in the development of endometriosis. Notwithstanding this evidence, various elements, such as the onset of random mutations, the disease phenotypic heterogeneity and the effective population disease prevalence, constitute a deep obstacle in understanding the underlying genetic mechanisms. In more recent years, genetic association studies but, mostly, genome-wide association studies (GWAS) have contributed to highlight genetic variants involved in the susceptibility to the disease [22,23]. The number of variants identified has increased over the years and, thanks to an international consortium, 27 genome-wide significant loci were found in association with endometriosis [13]. Notably, however, while the heritability of endometriosis has been estimated at 50%, the part explained by the genes identified so far in the field represents less than 10% [24].

A GWAS meta-analysis was carried out on 20,933 cases and 482,225 controls and a replication analysis was conducted on 58,115 cases and 733,480 controls. While some of the susceptibility single nucleotide polymorphisms (SNPs) identified were in or near genes related to cytoskeleton activity and matrix remodelling (rs1250247 near *Fibronectin 1*, rs495590 mapped in *Dynamin 3*, rs2510770 in *PDZ and LIM Domain 5*, rs71575922 in *Spectrin Repeat Containing Nuclear Envelope Protein 1*, rs7759516 in *Coiled-Coil Domain Containing 170*), hormonal response (rs74485684 in *Follicle Stimulating Hormone Subunit Beta*, rs11674184 in *Growth Regulating Estrogen Receptor Binding 1*, rs10012589 in *Kinase Insert Domain Receptor*) and regulation of growth factors (rs62468795 near *Insulin Like Growth Factor 2 mRNA Binding Protein 3*, rs17727841 in *Insulin Like Growth Factor 1*), some have been found to be involved in inflammatory processes or to be modulated in inflammatory pathways. More specifically, among genes observed overlapping or located near genome-wide significant loci, we can include *WNT4*, *IL-1A*, *VEZT* and *SKAP1*. The *Interleukin 1 Alpha* (*IL-1A*) gene encodes for a strong pro-inflammatory cytokine, member of the interleukin 1 cytokine family, produced by macrophages and monocytes as a proprotein. The proteolytic process is required to activate the protein after cell-injury, inducing apoptosis. In 2015, an association between *IL-1A* rs6542095 with moderate-to-severe endometriosis has been detected [25]. The *vezatin* gene (*VEZT*), encodes for the vezatin protein, widely expressed in the human endometrium and myometrium as part of the cadherin–catenin complex. Its expression rises during the secretory phase of the menstrual cycle. The *VEZT* promoter contains a binding site to nuclear factor kappa B (NF-kB) supporting a role for inflammatory factors in regulating VEZT-mediated activity [26]. The rs12320196 in *VEZT* had larger effect sizes for stage III/IV than stage I/II disease [12]. *SKAP1* gene encodes for a protein with immune-regulatory functions, including regulation of T-receptor signaling by enhancing the Mitogen-Activated Protein Kinase (MAPK) pathway and optimisation of conjugation between T-cells and antigen-presenting cells. The rs66683298 in *SKAP1* had similar associations with both stages I-II and III-IV endometriosis compared to controls [13].

According to some genetic association studies, several other polymorphisms of genes important in immune-regulation and inflammation have been reported to be linked to endometriosis. A correlation between *IL-16* rs4072111 and rs1131445 and disease progression has been detected. Interleukin-16 is a pro-inflammatory cytokine chemotactic for CD4+ T lymphocytes, monocytes and eosinophils [27]. The *TYK2* gene, encoding for the enzyme tyrosine kinase 2 (TYK2), which is part of the Janus kinase (JAK) family, also plays a critical role in both inflammation and autoimmunity. Its rs34536443 polymorphism appears to protect against endometriosis-related infertility, especially in advanced stages [28]. In contrast, the rs7528684 polymorphism of the *Fc-receptor like-3* (*FCRL3*) gene, which is involved in the activation of the NF-kB/MAPK pathways, increases the risk of disease-induced infertility, irrespective of disease stage [29]. Unfortunately, the role of these latter variants has not always been confirmed at genome-wide level. Table 1 recapitulates the main genetic variants involved in susceptibility to endometriosis that overlap or are located near genes involved in inflammatory processes or modulated by inflammatory pathways.

## 3. The MAPK Pathway

### 3.1. The MAPK Pathway between Genetics and Inflammation

In 2017, Uimari and colleagues presented, for the first time, a comprehensive analysis on the biological pathways involved in the pathogenesis of minimal/mild and moderate-severe stage endometriosis, using the GWAS technology and pathway-based analysis [30]. In general, GWAS investigates associations between common genetic variants and complex diseases, while pathway analysis is critical to detect the effects of GWAS variant data in genes, highlighting the pathogenic pathways involved [31]. Three significant pathways with genetic associations were described for any type of endometriosis; GRB2:SOS provides linkage to MAPK signaling for Integrins pathway, WNT signaling and p130Cas linkage to MAPK signaling for integrins pathway. The MAPK cascade has been described to be involved in the development of stage I-II endometriosis [29,32]. The rs144240142 variant, located in the intronic region of the *MAP3K4* gene was detected by GWAS analysis in association with mild disease (Table 1). Notably, MAP3K4 is part of the JNK and p38 MAPK pathways, whose role in the fine-tuning of inflammation and immune response has been revealed in different pathologies, such as rheumatoid arthritis [33], cardiovascular diseases [34], synaptic plasticity and neurodegeneration [35].

Recent studies have emphasized the importance of the MAPK cascade in the development and progression of endometriosis, due to its well-known involvement in the regulation of proliferation, gene expression, differentiation, mitosis, inflammation and survival/apoptosis [32,36,37]. MAPKs are part of the serine/threonine kinases and include three subfamilies that are distinguished by their biological function: Extracellular signal-Regulated Kinases (ERKs) [38] acting on the control of cell division, c-Jun N-terminal Kinases (JNKs) [39], involved in transcription regulation, and, finally, p38 mitogen-activated protein kinases (p38s) [40], that play a role in immune response, cell survival and differentiation. Their activation can occur due to a variety of stimuli, including mitogens, growth factors, oxidative stress and pro-inflammatory cytokines [41,42], and they require a receptor–ligand interaction to trigger the auto-phosphorylation cascade on their serine/threonine residues. The protein kinase cascade consists of enzymatic components, such as MAPKKK, MAPKK and MAPK, that are activated consecutively [43].

A detailed description of the studies that have evaluated the MAPK signaling in endometriotic lesions is presented elsewhere [37]. In general, this signaling has been suggested to favour the disease establishment via several mechanisms:

1. apoptosis [44] and angiogenesis [45], affecting cell growth;
2. migration [46] and invasion, possibly via a Transforming Growth factor ß-induced ERK activation through a Raf-dependent pathway [47];
3. production of inflammatory substances;
4. reactive oxygen species (ROS) production. The inflammatory environment typical of endometriosis is associated with alterations in ROS detoxification pathways and, consequently, determines an oxidative stress, both of which are involved in increased cell proliferation, particularly through activation of the MAPK ERK1/2 pathways [48];
5. progesterone resistance. Endometriosis is known to be characterized by a condition of progesterone resistance [49]. In particular, hyperactivation of the MAPK pathways is thought to suppress progesterone receptor activity via proteasome-dependent degradation, impairing endometrial decidualization and increasing the establishment of ectopic endometrial implants [50].

### 3.2. The MAPK Pathway as Therapeutic Target

Possible novel therapeutic approaches based on these findings may include the following.

#### 3.2.1. Inhibition of MAPKs

A MAPK ERK1/2 pathway inhibitor is Sorafenib, which has been shown to decrease the activity of this pathway by counteracting both endometriotic cell proliferation and neoangiogenic mechanisms in a xenogenic mouse model of endometriosis [51]. In 2006, FR 167653, an oral selective inhibitor of p38s MAPK, has been demonstrated to decrease experimentally induced endometriotic lesions in mice. In line for a role of MAPKs in inflammation, FR 167653 was able to reduce peritoneal inflammation and IL-6 levels [52]. Subsequently, the eicosanoid lipoxin A4 (LXA4), another p38s MAPK pathway inhibitor, has been shown to be useful in decreasing primary endometriotic stromal cells proliferation activity, migration and the endometriosis injury size in mouse models [53]. The JNK Inhibitor Bentamapimod (AS602801) has been shown to cause regression of ectopic endometriosis cells in vivo in rodent models, blocking the pro-inflammatory cytokine production at the site of injury [54]. Although the above treatments might be effective, induced adverse effects have to be weighted. Disease activity index and histological colitis score were significantly higher in FR 167653 treated mice [55]. In murine models, weight loss and impairment of reproductive functions (ovulation inhibition, embryotoxicity and teratogenicity) have been observed in case of MAPK inhibitors treatment [45].

#### 3.2.2. Inhibition of MAPKs and Progestins

Nowadays, oral, intramuscular, subcutaneous or intrauterine progestins are commonly used to treat endometriosis. They are synthetic molecules that can mimic the physiologic activity of progesterone, reducing estrogen and inflammation levels [56]. The potential role of MAPKs in inducing progesterone resistance would suggest the use of progestin treatment in combination with MAPK inhibitors for the treatment of endometriosis [50].

**Figure 1:**
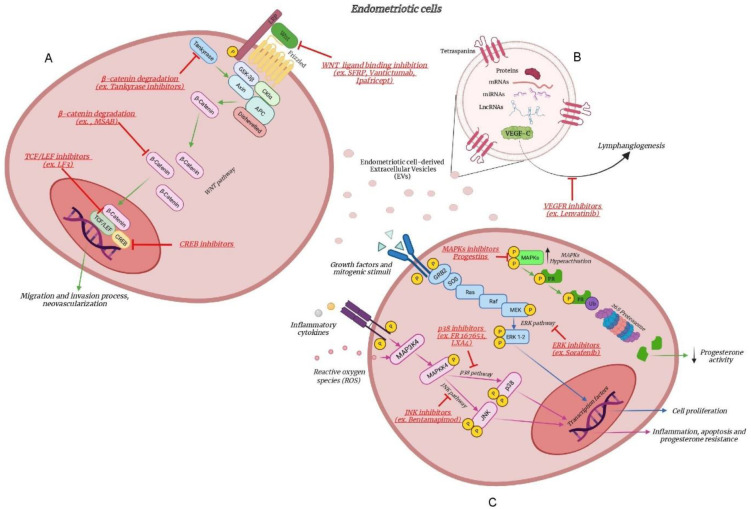
Schematic representation of the WNT pathway (**A**) and MAPK pathways (**B**) responsible for cell proliferation, migration, neovascularization, inflammation and progesterone resistance in endometriotic cells. Magnified representation of a VEGF-C-carrying extracellular vesicle (EVs) (**C**) involved in enhancing the lymphangiogenic capacity of lymphatic endothelial cells. Next-generation inhibitors that could be used to treat patients with endometriosis are shown in red and directed toward their specific therapeutic target. Created with BioRender.com.

These options have been described in Figure 1.

## 4. The WNT Pathway

### 4.1. The WNT Pathway between Genetics and Inflammation

Genome-wide association studies on endometriosis have demonstrated an association between susceptibility to the disease and markers located in or near the WNT ligand *WNT4* gene encoding for a noncanonical key player in the development of the female reproductive tract [57]. Mafra et al. studied the relationship between endometriosis and four *WNT4* SNPs, suggesting that two SNPs (rs16826658 and rs3820282) of the *WNT4* gene might be involved in the pathogenesis of endometriosis in infertile women (Table 1) [58]. It is however still unclear whether these SNPs affect gene transcription. Genome-wide enrichment analysis allowed Rahmiouglu et al. not only to confirm the association of WNT4 to endometriosis but also to figure out novel susceptibility loci. Overrepresentation of other variants of the WNT pathway (rs560584 near *KIFAP3*) and a stronger enrichment in cases of more severe disease have been documented [59].

The WNT signaling pathway plays essential roles in tissue or organ homeostasis by regulating cell proliferation, differentiation and tissue regeneration [60]. Upon tissue or organ injury, inflammation is coupled with tissue repair and regeneration process. Some of the members of the pathway have been identified as pro-inflammatory ligands and their expression levels have been associated with a more severe inflammatory process [61]. The WNT signaling comprises different pathways whose central players can be β-catenin and WNT ligands, endogenous β-catenin inhibitors, such as secreted frizzled-related protein (sFRPs), and members of the Dickkopf family (DIKK).

A higher expression of WNT4 mRNA has been demonstrated in the eutopic endometrium of women with endometriosis than in that of healthy women, along with WNT2 and DKK1 [62]. Up-regulated mRNA levels of sFRP2 were described in ectopic tissue compared to eutopic endometrium [63]. Similarly, Heinosalo et al. proved that sFRP2 localizes in both epithelium and stroma of extraovarian endometriosis tissue along with β-catenin promoting cell proliferation. Moreover, the authors assumed a negative correlation between sFRP2 mRNA expression with intracellular progesterone concentration, as two progesterone responsive elements were identified in the sFRP2 promoter. This result strengthens the hypothesis of a disturbed hormonal environment as a central upstreaming mechanism of WNT signaling in endometriosis [64].

In line with this evidence, an intense crosstalk between estradiol and WNT/β-catenin signaling could be postulated for the establishment of this disease requiring cell migration, invasion and neovascularization [65,66,67]. More specifically, this signaling has been suggested to affect the disease establishment via several mechanisms:

1. migration and invasion process. Xiong et al. studied the effect of xeno-transplantation of human endometrium cells into NOD-SCID mice under estradiol treatment [65]. They observed that abnormal levels of estradiol were capable of inducing β-catenin expression in a dose- and time-dependent manner through the involvement of estrogen receptor (ER)-α. Moreover, along with previous evidence by Becker et al. in 2010, the authors showed that the estradiol-dependent increased level of β-catenin could up-regulate the production of MMPs, such as MMP-9, involved in extracellular matrix remodelling, allowing cellular migration and invasion [68].
2. neovascularization. In 2008, Cheng et al. showed that the sFRP1 transcript, already known for its proangiogenic effects [69], was higher in the proliferative phase endometrium and significantly increased in endometriotic tissues compared with eutopic endometrium [69]. In human endometrial stromal cells, Zhang et al. proved that the regulation of vascular endothelial growth factor (VEGF) by estradiol involved the multifunctional protein β-catenin and the transcription factors TCF3/LEF1 [67]. In detail, at a genomic level, the binding of ERα to estrogen responsive elements in the β-catenin promoter induced its transcription, on one hand; on the other hand, aberrant estrogen levels inactivated GSK3β, preventing β-catenin from its degradation. Both ways converged into the enhanced level of β-catenin in the nucleus, modulating the expression of VEGF [67]. VEGF is thought to be a crucial factor in endometriosis establishment, as ectopic and eutopic endometrium of endometriosis patients have high VEGF mRNA levels [70].
3. production of inflammatory cytokines. Jiang and coworkers suggested that the anti-inflammatory cytokine IL-37 suppressed the proliferation, adhesion, migration and invasion of human ectopic endometriotic stromal cells from ovarian endometrioma samples, through multiple signaling pathways, with the β-catenin pathway as one of the most critical. IL-37 overexpression significantly suppressed IL-1β, IL-6, IL-10 and and TNF-α, whereas knockdown of IL-37 significantly upregulated their protein and mRNA expression, compared to the control. From the in vivo experiment, the lesion size of endometriosis mice treated with recombinant IL-37 was significantly decreased, compared with the control mice [71].

Notably, a relationship between MAPK and WNT signaling has already been depicted, although the nature of the interaction seemed to be strictly connected to specific cellular context [72,73]. Despite the apparent contrasting crosstalk in case of melanoma compared to colorectal cancer, axin, one of the destruction complex components of β-catenin has been suggested as a key node for coordination between the two pathways [74]. However, additional work is required as there is no direct evidence of the interplay in case of endometriosis.

### 4.2. The WNT Pathway as Therapeutic Target

The comprehension of the relationship between endometriosis and the WNT/β-catenin pathway may open the venue for new possible therapeutic strategies (Figure 1). WNT/β-catenin signaling-targeted interventions have already been investigated in malignancies such as colorectal cancer, breast cancer and leukaemia [75]. Despite the high number of targets, we will briefly sum up the three main mechanisms that could be intriguing in endometriosis field [76].

#### 4.2.1. β-Catenin Degradation

In preclinical trials, Tankyrase inhibitors could induce the protein degradation complex, down regulating WNT signaling [77]. Indeed, one of the molecules that determines β-catenin degradation, AXIN, is destroyed by Tankyrase ubiquitination. Similarly, the compound methyl 3-[(4-methylphenyl)sulfonyl]amino-benzoate (MSAB) could accelerate the proteasomal degradation of β-catenin through its ubiquitination [78].

#### 4.2.2. Inhibition of WNT Ligand Binding

SFRP peptides induce WNT pathway inactivation, blocking the binding of SFRP to WNT ligands, due to their structural homology [79]. More recently, monoclonal antibodies and recombinant fusion proteins have been designed. Vantictumab (OMP-18R5; NIH clinical trial numbers (clinicaltrials.gov); NCT02005315, NCT01973309, NCT01345201 and NCT01957007), binding to FZD receptors, prevents them from interacting with WNT ligands [80]. Ipafricept (OMP-54F28; NIH clinical trial numbers: NCT02069145, NCT02050178, NCT02092363 and NCT01608867) could compete with FZD receptors, binding to WNT ligands [81].

#### 4.2.3. Transcriptional Activity Inhibition

Transcription complex and co-activators could be another fruitful target. Preclinical studies have used inhibitors of TCF/LEF and co-activators [82]. LF3, a sulfonamide derivative, reduces the interaction between β-catenin and TCF4 [83]. CREB binding proteins are essential co-activators for transcription, so corresponding inhibitors have been considered [84,85].

However, some severe side effects for these treatments have already been described. An important dose-limiting toxicity in case of Tankyrase inhibitors derives from the impairment of bone biology, as observed in mice [86]. Similarly, bone toxicity prevents Ipafricept from the continued development for epithelium ovarian cancer [81].

Few data related to Vantictumab clinical effects are available [87]. Overall, antagonizing the WNT pathway could profile as a great challenge for a double reason. On one hand, the complexity and redundancy of the pathway obstacles the introduction of a single-target therapy. On the other, the WNT/β-catenin cascade globally presides over tissue homeostasis and has pleiotropic effects, so that it is not hard to expect main side effects from related inhibitors [88,89].

In this regard, unravelling the interplay between the WNT and MAPK cascades could facilitate the development of combined therapeutic protocols for endometriosis, as already figured out for other cancers [90].

## 5. The Extracellular Vesicles Modulating Inflammation

### 5.1. The Extracellular Vesicle Cargo as Therapeutic Target

Extracellular vesicles (EVs) have been described as an extraordinary tool for future applications in both diagnosis and therapy [91]. Owing to their high delivery efficiency, biocompatibility and multifunctional properties, EVs are expected to become a new means of drug delivery, disease diagnosis, immunotherapy and precise treatment. The relationship between number, size, content (DNA, RNA and proteins) of these nanoparticles and the progression of different diseases are rapidly emerging [92]. Immunomodulatory functions of EVs are now widely recognized in several physiological conditions, including pregnancy and pathological scenarios, such as various cancers and chronic inflammatory diseases, with potential implications in the development of novel diagnostic and therapeutic modalities [93,94].

Recently, the molecular profile and the potential functions of EV-cargo in endometriosis have been reported. Patients with endometriosis showed higher levels of vascular endothelial growth factor (VEGF)-C carried by EVs into the bloodstream throughout the body [95]. Li and coworkers demonstrated that VEGF-C-carrying EVs derived from endometriotic stromal cells were able to improve the lymphangiogenic capacity of lymphatic endothelial cells. The formation of lymphatic vessels provides additional pathways for immune cells to infiltrate endometriotic tissues and to secrete proinflammatory cytokines. The authors suggested a molecular mechanism of lymphangiogenesis and its role in the pathogenesis of endometriosis where proinflammatory cytokines (IL-1β and TNF-α) suppress the expression of COUP-TFII in endometrial stromal cells that cause VEGF-C- secretion through EVs. Carried by EVs, a stable and effective means of transport, VEGF-C reaches sentinel lymph nodes and binds to VEGFR2/3 on lymphatic endothelial cells to induce lymphangiogenesis towards endometriotic lesions. This feed-forward loop may explain how the lymph-attractant produced by endometriotic cells may reach the lymphatic endothelial cells that are far from the endometriotic lesions. The involvement of VEGF-C carried by EVs would not only be useful as a biomarker from a diagnostic point of view but could be considered as a potential molecular target for the development of therapeutic regimens. Indeed, in the same study, the infiltration of immune cell populations (macrophages, regulatory T cells and Th17 lymphocytes) into the endometriotic lesions could be significantly reduced by treatment with Lenvatinib, a selective inhibitor of VEGFR2/3. Lenvatinib was shown not only to decrease lesion size in a mouse model of endometriosis, but also to reduce the number of lymphatic vessels and inflammation parameters. The authors concluded that Lenvatinib could inhibit lymphangiogenesis blocking the action of VEGF-C carried by EVs. Lenvatinib is a promising drug for treating cancers and is already in use to treat thyroid cancer and metastatic renal cell carcinoma. Clearly, the use of this drug in the treatment of endometriosis is not acceptable due to its numerous side effects (i.e., high blood pressure, proteinuria, hand-foot syndrome, abdominal pain and dysphonia) [96]. However, since its aim in endometriosis treatment is to inhibit the action of VEGF transported by EVs, a different targeted anti-VEGF-EV therapy could be envisaged. An EV-targeted therapy approach could be implemented, for example, through the use of immunoconjugates, consisting of antibodies capable of recognizing specific enriched EV antigens (ligands, receptors, proteins, etc.), a VEGF inhibitor (such as a soluble VEGFR) [97] and a linker that would ensure that the effector does not separate from the antibody. The use of immunoconjugates would ensure the delivery of therapeutic agents to specific targets (EVs), avoiding the problems associated with the systematic administration causing unacceptable host toxicity [98].

### 5.2. The Extracellular Vesicles Cargo as Therapeutic Tool

Extracellular vesicle-associated molecules may be not only interesting as potential biomarkers of endometriosis progression, but they may also serve as therapeutic regimens per se (Figure 1). Exosomes have the potential to be a useful therapeutic tool for various diseases, such as cardiomyopathy, cancer and neurodegenerative diseases [99]. The use of EVs possesses numerous advantages over cell-based therapies, especially in the context of regenerative medicine, such as less restriction associated with safety, and feasibility of canonical cell transplantation, such as cell engraftment, immunocompatibility and survival [100]. The current “state of the art” of EV fractions used as a therapeutic agent can be evidenced by several active clinical trials. They include an early phase-1 clinical trial using adipose-derived stem cell exosomes for the treatment for periodontitis (ClinicalTrials.gov Identifier: NCT4270006) or a phase-2 clinical trial (ClinicalTrials.gov Identifier: NCT01159288) to evaluate the ability and safety of autologous dendritic cell-derived exosomes, loaded with tumor antigens, in activating tumor-specific cytotoxic T cells as a vaccination in lung cancer patients [101].

Extracellular vesicles could be a suitable therapeutic tool thanks to the crosstalk via RNA, due to the fact that EV-RNAs, being protected from RNAses, may better maintain their functional integrity. This is the case of exosomal miR-214-3p, able to reduce the fibrosis level when it is used to treat endometriosis in mouse models [102].

Fibrosis of ectopic lesions represents a critical feature of endometriosis [103]. Zhang and collaborators identified connective tissue growth factor (CCN2) as an important mediator in lesion fibrosis. Being a downstream target of transformation growth factor-β (TGF-β), CCN2 is linked to fibrosis in many tissues and diseases [104,105] and represents an attractive therapeutic target whose expression can be regulated by several miRNAs. In particular, the authors found that, in a mouse model of endometriosis, miRNA-214-3p can regulate fibrotic proteins by affecting the miR-214-3p–CCN2 axis. Their experiments have supported the inhibitory effect of miR-214-3p on CCN2 expression and, consequently, on the levels of fibrogenesis, both in vitro and in vivo. Evidence in vitro indicated that mir-214-3p is transferred by exosomes and is biologically active upon its absorption by recipient cells. More specifically, the exosomal delivery of miRNA-214-3p into ectopic endometrial stromal cells caused a decrease in endogenous CCN2 mRNA expression. In vivo, intraperitoneal injection of exosomes purified from supernatants of cells previously transfected with miRNA-214-3p mimics, reduced the expression of fibrosis-related proteins (CCN2, α-SMA and collagen a1) in endometriotic lesions. To note, intraperitoneal injection of “free” miRNA-214-3p alone did not cause the same fibrosis-inhibiting effect, supporting the idea that mir-214-p-associated with exosomes can be considered as a new potential therapeutic treatment for the management of the disease and its progression.

On the basis of the findings by Zhang et al., the group of Wu and collaborators defined a new EV-function based on competing endogenous RNA (ceRNA) in endometriosis [106]. The ce-RNA theory proposes that RNA species could regulate each other by competing with miRNA response elements. Specifically, lncRNAs may reduce the bioavailability of a specific miRNA by acting as a molecular sponge, containing multiple binding sites for the specific miRNA and preventing it from binding/inhibiting its target mRNA [107,108]. In this context, the exosomal lncRNA–miRNA–mRNA ceRNA regulatory networks have been defined in endometriosis using weighted gene co-expression network analysis (WGCNA) algorithms. A new mechanism has been identified in endometriosis, involving the lncRNA LOC105376166 (more abundant in ectopic cell-derived EVs than in those from normal endometrial stromal cells) that could promote the expression of Mindbomb E3 Ubiquitin Protein Ligase 2 (MIB2) by competing to the shared binding by miR-214-3p in exosomes. MIB2 is differentially expressed in various endometriotic lesions and regulates the endometriosis-associated Notch signaling pathway and the NF-kB cascade [109]. Thus, it may lead to the release of more MIB2 from the endometrial stromal cells to the receipt cells in the uterine or the abdominal cavity and to an additional contribution to the pathogenesis of endometriosis. This evidence provides a novel view on the RNA–RNA crosstalk via EVs and indicates the potential diagnostic and therapeutic roles of exosomal ceRNA networks in endometriosis [106].

Similarly to lncRNA–miRNA interaction, two other axis networks mediated by exosomes have been suggested by Khalaj et al. in endometriosis [94]. The authors identified three miRNAs, miR-30d-5p, miR-27a-3p and miR-375, unique to endometriosis, which were exclusively present in exosomes isolated from both serum and culture supernatant of ectopic lesions collected from endometriosis patients. In the same study, lncRNA signatures of EVs from ectopic and eutopic endometrium and endometrial tissue from healthy controls have been compared. Among the differentially abundant LncRNAs, LncRNAs H19 and NEAT1 resulted less abundant in ectopic lesion-derived EVs than in EVs from normal endometrium of healthy controls. Both of these lncRNAs have been previously implicated in endometrial pathologies involving cell proliferation of stromal cells via insulin growth factor signaling, as well as invasive and migratory abilities [110,111]. They also shared binding sites with at least two of the aforementioned miRNAs. Additionally, the authors reported that many cytokines and signaling pathway genes implicated in endometriosis, as well as inflammation and angiogenesis, were regulated by the same miRNAs in the network (IL-1A, IFNG, EGFR, ERBB2, MAP3K8, JAK2, STAT3, NOTCH1, YAP1 and IRF3). To support the involvement of these lncRNA–miRNA networks in angiogenic and inflammatory mechanisms, Khalaj et al. aimed to identify the functional effect of EVs derived from endometriotic cells (12Z) treating endothelial cells (HUVECs) with endometriotic epithelial cell-derived EVs. The stimulation of angiogenic and inflammatory factors confirmed by the presence of G-CSF, TNF-α and PDGF-AA cytokines was demonstrated in the spent media of endothelial cells co-cultured with endometriotic epithelial EVs [94].

At the current laboratory stage, EVs—especially exosomes—could possibly replace current exogenous nanocarriers to reduce toxicity and immunogenicity to a great extent [112]. Human EVs may act as vectors in RNA-based gene therapy for the treatment of endometriosis. The fact that exosomes can be engineered to express foreign proteins, miRNA and also siRNA and to carry drugs allows not only the expression of proteins/drugs delivery in a tissue-specific manner, but also the silencing of specific genes [99].

The possibility to introduce chemical or biological modifications in EVs in order to supplement or expand their therapeutic application enhances the potential of these nanoparticles as intelligent nanotherapy technology. Taking exosomal therapy from the laboratory bench to the clinical stage is an important step forward but, as a new type of drug delivery system, the safety of exosomes must be given sufficient attention in the process of clinical application.

## 6. Conclusions

Thanks to experimental models of endometriosis, we have learnt a lot about the development of endometriosis in terms of the steroid responsiveness, inflammatory process and peritoneal environment related to the disease. As the knowledge of the disease increases, new therapies may develop in order to alleviate symptoms, stop the progression, preserve fertility and reduce the likelihood of recurrence. Information deriving from the studies at the intersection between inflammation and genetics are important not only for the analysis of molecular and cellular mechanisms underlying the disease pathogenesis but also for the development of novel therapeutic strategies. These studies have revealed the importance of signaling pathways, such as MAPK and WNT, known to be involved in activities such as migration, cell survival, growth and angiogenesis, which are critical for the progression of the disease. Indeed, genes involved in inflammatory pathways, such as WNT4 and MAP3K4, have been observed overlapping or located near loci involved in the susceptibility to the disease. Since these pathways are also important for the crosstalk with the immune system elements, it is also possible to gain insight into the microenvironment that they contribute to support. In this context, the identification of EVs with a potential role in influencing the endometriosis environment acquires a particular meaning. Extracellular vesicle-associated molecules may not only be interesting as potential biomarkers of endometriosis progression, but they may serve as therapeutic regimens per se. As the role of EVs in endometriosis is complex, a higher level of complication may be introduced with novel factors involved in the process of endometriosis potentially targeting new therapeutic strategies.

## Figures and Tables

**Table 1:** Genes involved in inflammatory processes or modulated by inflammatory pathways observed overlapping or located near loci involved in the susceptibility to endometriosis.

Pathways	Genes	SNPs
*Inflammation*	*IL1A*	rs6542095
	*VEZT*	rs12320196
	*IL16*	rs4072111; rs1131445
	*SKAP1*	rs66683298
	*TYK2*	rs34536443
	*FCRL3*	rs7528684
	*MAP3K4*	rs144240142
	*WNT4*	rs16826658; rs3820282
